# Time-dependent cytotoxic drugs selectively cooperate with IL-18 for cancer chemo-immunotherapy

**DOI:** 10.1186/1479-5876-9-77

**Published:** 2011-05-25

**Authors:** Ioannis Alagkiozidis, Andrea Facciabene, Marinos Tsiatas, Carmine Carpenito, Fabian Benencia, Sarah Adams, Zdenka Jonak, Carl H June, Daniel J Powell, George Coukos

**Affiliations:** 1 Ovarian Cancer Research Center, School of Medicine, University of Pennsylvania, Philadelphia, USA; 2 Abramson Family Cancer Research Institute, School of Medicine, University of Pennsylvania, Philadelphia, USA; 3 Department of Pathology and Laboratory Medicine School of Medicine, University of Pennsylvania, Philadelphia, USA; 4 GlaxoSmithKline, King of Prussia, USA

## Abstract

**Background:**

Time-dependent chemotherapeutic agents can selectively target tumor cells in susceptible phases of the cell cycle however a fraction of tumor cells in non-vulnerable cell cycle phases remain drug-resistant. Immunotherapy represents a promising approach to overcome the limitation of phase-specific drugs and improve their clinical efficacy. Here, we investigated the potential use of anticancer chemotherapeutic drugs in combination with IL-18, a cytokine with strong immunostimulatory properties.

**Methods:**

Four chemotherapeutic drugs commonly used in ovarian cancer were first tested for the ability to increase the immunogenicity and killing of the murine ovarian cancer cell line ID8 in vitro. Chemotherapeutric agents with measured time-dependent immune-enhancing effects were then tested for antitumor effectiveness in vivo in combination with IL-18 immunotherapy using the ID8-Vegf ovarian cancer model.

**Results:**

Paclitaxel or topotecan exposure alone mediated incomplete, time-dependent killing against the murine ovarian cancer cell line ID8 in vitro, whereas carboplatin or gemcitabine mediated comprehensive, dose-dependent killing. In the plateau phase of the time-dependent killing by topotecan or paclitaxel, drug-resistant ID8 cells were more immunogenic with elevated expression of MHC-I and Fas, and increased sensitivity to CTL and Fas agonistic antibody in vitro. Moreover, the antitumor effectiveness of time-dependent agents *in vivo *was significantly improved with the addition of IL-18 through a T cell-dependent mechanism, while the effectiveness of drugs without significant phase specificity were not.

**Conclusions:**

Tumor immunotherapy with IL-18 can significantly augment the killing fraction of phase-specific chemotherapeutic drugs and provide survival benefit. The safety profile of IL-18 and its positive interactions with select anticancer chemotherapeutic agents strongly supports the clinical investigation of this combinatorial approach.

## Background

Although chemotherapy is the treatment of choice for many types of cancer, it is rarely curative in most solid tumors. Immune therapy represents a potentially attractive approach to increase the efficacy of chemotherapy by targeting cancer cells that escape chemotherapy. However, it has been unclear to date whether any chemotherapy drugs are more suitable than others for such combinations, and empirical use has produced mixed results. For example, although higher objective response and disease control rates, along with elevated frequencies of cytolytic tumor antigen-specific T cells, were seen in patients with metastatic colorectal carcinoma receiving polychemotherapy with gemcitabine plus oxaliplatin, fluorouracil, and folinic acid (FOLFOX-4) followed by granulocyte-macrophage colony-stimulating factor (GM-CSF) and low-dose interleukin-2 (IL-2) [1], addition of IL-2 and interferon-alpha2b did not increase the efficacy of cisplatin, vindesine and dacarbazine in melanoma patients [2]. Thus, understanding the mechanisms underpinning positive chemo-immunotherapy interactions is a critical task for the development of effective cancer therapy.

Previous reports have suggested that the exposure of tumor cells to chemotherapeutic drugs can sensitize them to immune effector cells [3-6]. Theoretically, to achieve synergy with immune therapy and increased tumor killing, chemotherapy should sensitize to immune killing tumor cells that are destined to survive chemotherapy. Depending on their mechanism of action, the efficacy of chemotherapy drugs may be influenced markedly by the time of exposure (phase-specific or time-dependent drugs) or by the dose that can be administered (phase-nonspecific or dose-dependent drugs). The efficacy of phase-specific anticancer drugs is time-dependent, as only a fraction of tumor cells are in appropriate cell cycle phase for chemotherapy-mediated killing at any given time. Thus, a fraction of tumor cells remains alive following administration of each chemotherapy dose and can eventually repopulate the tumor following completion of chemotherapy [7-10]. We hypothesized that because of this property, time-dependent chemotherapy drugs are more likely to benefit from combination with immune therapy.

Interleukin 18 (IL-18) is a pleiotropic cytokine, originally described as interferon (IFN)-γ inducing factor, that can mediate immunostimulatory effects on immune cells of the adaptive and innate immune system [11]. Its multiple immunologic activities include the induction of IFN-γ, TNF-α, IL-1α, and GM-CSF production; augmentation of natural killer (NK) cell cytotoxicity; and promotion of Th1 differentiation of naive T cells. These features render IL-18 an interesting candidate for tumor immunotherapy. As a single agent, IL-18 was shown to elicit anti-tumor reactivity when administered at high doses in mice with established tumors [12]. The immunostimulatory activity of IL-18 *in vivo *has been demonstrated in non-human primates [13] and humans [14]. In phase I clinical evaluation, recombinant human (rh)IL-18 was safely administered as monotherapy to 28 patients with solid tumors, with minimal dose-limiting toxicities and two partial tumor responses [14]. Toxicity has generally been mild to moderate even with repeat administration and a maximum tolerated dose has not been reached to date [15]. IL-18 enhanced activation of peripheral blood CD8^+ ^T cells, NK cells and monocytes and induced a transient increase in the frequency and expression level of Fas ligand (FasL) in peripheral blood CD8^+ ^T cells and NK cells [15]. The relatively minor toxicity of rhIL-18, compared with other immunostimulatory cytokines that have undergone clinical development, is remarkable and renders IL-18 a well suited drug for combinatorial approaches with chemotherapy.

In the current study, we characterized the immune effects on tumor cells of four common anticancer chemotherapy drugs utilized in ovarian cancer and other solid tumors, two phase-specific (time-dependent) drugs, paclitaxel and topotecan, and two phase-nonspecific (dose-dependent) drugs, gemcitabine and carboplatin. Both paclitaxel and topotecan exert their actions on dividing cells, acting as phase-specific chemotherapeutic drugs. Paclitaxel inhibits the dissolution of microtubules, enhances tubulin polymerization and produces a block in the metaphase of mitosis, leading to growth inhibition and cell apoptosis [16]. Topotecan, a topoisomerase I inhibitor, stabilizes the covalent complex of enzyme and strand-cleaved DNA, which is an intermediate of the catalytic mechanism, thereby inducing breaks in the protein-associated DNA single-strands, resulting in cell death [17]. Carboplatin is a classic cycle phase non-specific drug [18]. The main mechanism of action of gemcitabine is inhibition of DNA synthesis. The killing effects of gemcitabine are however not confined to the S-phase of the cell cycle and the drug is equally effective against confluent cells and cells in log-phase growth [19]. Incorporation of gemcitabine into RNA is another action, which is time- and concentration-dependent and leads to inhibition of RNA synthesis. In human tumor cell lines displaying different degrees of resistance to gemcitabine, sensitivity to this drug was related to differences in RNA incorporation [20]. Moreover, several metabolites of gemcitabine can inhibit various enzymes, leading to self-potentation of gemcitabine action [21]. Thus, the overall mechanism of action of gemcitabine is phase non-specific.

Because the effect of immune therapy becomes clinically relevant only if immune mechanisms target the tumor fraction surviving chemotherapy, we focused on the fate of tumor cells escaping direct killing by chemotherapy. We hypothesized that these cells are sensitized to immune therapy, which enables a cooperation between immunotherapy and time-dependent (phase-specific) drugs. Thus, we hypothesized that among chemotherapy drugs, time-dependent (phase-specific) drugs are more likely to benefit from IL-18 therapy combination. We investigated these interactions in a mouse model of ovarian cancer [22]. IL-18 alone had a modest antitumor effect, while it exhibited positive interaction with select chemotherapeutic drugs, improving their therapeutic effect *in vivo*. Chemotherapeutic agents upregulated immune molecules in tumor cells and sensitized them to immune-mediated killing. Importantly, this effect was translated to a significant increase in total killing fraction and better outcome only for time-dependent drugs. Interestingly, combination of IL-18 with dose-dependent drugs did not increase their efficacy *in vivo*. In this study however we sought to mainly explore the ability of those drugs to render tumor cells more susceptible to immunotherapy with IL-18, and specifically focused on effector mechanisms, and not the drugs' overall effects on the immune system. Moreover, our findings indicate for the first time that the difference of chemotherapeutic drugs in their ability to interact with immunotherapy might be attributed to their mechanism of action directly on the tumor cell. These data suggest that tumor immunotherapy with IL-18 may potentiate the antitumor effect selectively of time-dependent chemotherapeutics used in various types of cancer.

## Methods

### Cell Culture and Reagents

ID8 ovarian cancer cells were generously donated by Drs. Kathy Robby and Paul Terranova (Kansas University) [23]. The development and characterization of ID8-Vegf cell line was described elsewhere in detail [22]. The development and characterization of the ID8-E6E7 cell line transfected to stably express the E6 and E7 genes of the human papilloma virus was described elsewhere in detail [24]. Briefly, ID8 cells were transduced with the retroviral vector LXSN16E6E7 (American Type Culture Collection, Rockville, MD, donated by Dr. D. Galloway), which encodes the HPV16 E6 and E7 genes, as well as the neomycin phosphotransferase gene. The PA317 cell line was used to generate the retroviral vectors as previously described [25]. Selection of ID8 cells transduced with E6 and E7 (ID8-E6/7) or ID8 cells transduced with a control retroviral vector (LXSN) was achieved under neomycin pressure (1mg/ml). ID8, ID8-E6E7 and ID8-Vegf cells were maintained in DMEM media (Invitrogen, Carlsbad, CA) supplemented with 10% fetal bovine serum (FBS), 100 U/ml penicillin, and 100 μg/ml streptomycin (Roche, Indianapolis, IN) in a 5% CO_2 _atmosphere at 37°C. ID8-Vegf cells were used for *in vivo *experiments. ID8 cells were used for *in vitro *and flow cytometry experiments. ID8-E6E7 cells were used as target cells for cytotoxicity assays. All reagents were from Sigma unless stated otherwise.

### Mice and Treatments

Eight to sixteen week old female C57BL/6 mice and C57BL/6 severe combined immunodeficient (SCID) mice (Charles River Laboratories, Wilmington, MA) were used in protocols approved by the Institutional Review Board of the University of Pennsylvania. Mice were treated with intraperitoneal (i.p.) bolus injections of chemotherapy drugs or PBS (controls) as follows: Carboplatin, 20 mg/kg in 0.2 ml 0.9% saline, four weekly doses; paclitaxel, 15 mg/kg in 0.3 ml 0.9% saline, four weekly doses; topotecan, 2.5 mg/kg in 0.2 ml 0.9% saline, every 5 days (total five doses); and gemcitabine, 25mg/kg in 0.2ml 0.9% saline, every 3 days (total five doses). Chemotherapy doses were approximately one-fourth to one-sixth of the respective maximally tolerated dose (MTD) for mice [26-30]. Recombinant murine (rm)IL-18 (GlaxoSmithKline) was given s.c daily for 40 days at 10 μg/mouse in 90 μl PBS. Control mice received s.c. daily injections of PBS (100 μl) alone. Chemotherapy administration was started 8 days after tumor inoculation and IL-18 treatment was started two days later.

### Tumors

Orthotopic intraperitoneal (i.p.) tumors were generated by inoculating intraperitoneally 5 × 10^6 ^ID8-Vegf cells suspended in 250 μl PBS. Mice were weighed weekly starting two weeks after tumor inoculation. Ascites volume measurement was a terminal procedure and it was done when mice reached 35 grams of weight. When we needed week-to-week measurements of ascites accumulation, we used weight increase as surrogate. Solid subcutaneous tumors were generated by inoculating 10^7 ^ID8-Vegf cells suspended in 250 μl PBS and mixed with an equal volume of cold Matrigel into the flanks of mice. Tumors were detectable two weeks later and tumor size was measured weekly thereafter using a Vernier caliper. Since studies have shown that tumor weight is the most consistent and reproducible reflection of tumor volume, especially in small tumors [31], tumor volumes were calculated by the formula V = ½(L × W)^2^, where L is length (longest dimension) and W is width (shortest dimension). Experiments were terminated when control tumors reached the size of 600 to 800 mm^3^; all groups were euthanized, the tumors were excised and weighed.

### In vitro treatment of tumor cells

For measurement of chemotherapy sensitivity in vitro, ID8 tumor cells (1.3 × 10^5^/well) were exposed to cytotoxic agents for 6 hours in various concentrations: Topotecan (0, 0.1, 0.3, 1, 3, 10, and 30 μg/ml), paclitaxel (0, 0.03, 0.1, 0.3, 1, 3, and 10 μg/ml), gemcitabine (0, 0.02, 0.06, 2, 6, 20, and 60 μg/ml), carboplatin(0, 30, 100, 300, 1000, and 3000 μg/ml) and to sodium azide (0, 0.1, 0.3, 1, 3, and 10 μg/ml). At 6 hours the drug containing media was removed, cells were washed twice with PBS and cultured in drug free media for another 42 hours. Tumor cells were then washed twice with PBS, thrypsinized and counted. Non-viable cells were excluded with Trypan Blue staining. For Fas-mediated killing, we used the anti-Fas agonistic antibody, Jo2 (0.2ug.mL dose, BD PharMingen). Protein G (2 μg/ml; Biovision) was used to maximize cross-linking of the primary antibody. The antibody was added 24 hours before cell harvesting and counting. Control wells consisted of tumor cells incubated with isotype-matched antibody and protein G. ID8 cell survival fractions after exposure to different doses of drugs with or without exposure to Fas agonistic antibody were plotted against the drug concentrations and the resulting data set was fit to a logistic dose-response function using Origin7 software (OriginLab Corporation). IC_50 _values were obtained from the fit parameters that achieved the lowest *x^2^*value.

### Flow cytometry analysis

Cell surface staining of mouse splenocytes was performed with FITC-labeled anti-CD69 mAb, PE-labeled anti^_ ^CD4 mAB, Pe-Cy7-labeled anti-CD3 mAB, PercP-labeled-anti-CD8 mAb. For the cell surface staining of ID8 tumor cells anti-MHC-I (H-2Kb/H-2Db) biotinylated mAb and anti-Fas PE-labeled mAb were used. Secondary Ab was streptavidin-APC labeled. All mAb were purchased from BD PharMingen. Cell fluorescence was analyzed and compared with the appropriate isotype-matched controls (BD PharMingen) with a FACSCanto cytometer and Flow Jo software.

### Apoptosis detection

Detection of apoptotic cells was carried out with the TACS Annexin V-FITC apoptosis detection system (R&D Systems) which uses annexinV-fluorescein isothiocyanate (FITC) conjugates for flow cytometry.

### Lymphocyte cytotoxicity assay

ID8-E7 cells were prepared for use as targets in a colorimetric non-radioactive cytotoxicity assay measuring lactate dehydrogenase (Promega). Target cells were exposed to topotecan or paclitaxel for 6 hours, washed and cultured for an additional 42 hours. The target cells (12 × 10^3 ^cells/well) were coincubated with splenocytes from vaccinated donor mice (see below) at various effector:target (E:T) cell ratios, in a final volume of 200 μl RPMI supplemented with 10% FBS (100 U/ml penicillin and 100 μg/ml streptomycin) for 4 hours at 37°C with 5% CO_2_. For the generation of effector cells, eight to sixteen week old C57BL6 mice were vaccinated twice, one week apart, with LLO-E7 DNA vaccine [32] encoding the E7 peptide and the Listeria Listeriolysin O (LLO) adjuvant (kindly provided by Dr. Yvonne Patterson), and one month later they were challenged with 5 × 10^5 ^TC-1 cells (which express E7) injected s.c in the flank as boost, which increases significantly the frequency of E7-reactive T cells in the spleen. Two weeks later, mice were sacrificed, spleens were harvested, splenocytes were isolated and stimulated *in vitr*o for 7 days with 30 IU/ml IL-2 and 8 μg/ml E7 peptide in RPMI media (Invitrogen) supplemented with 10% fetal bovine serum (FBS), 100 U/ml penicillin, and 100 mu μg/ml streptomycin (Roche) in a 5% CO2 atmosphere at 37°C. The percent cytotoxicity was calculated with the formula:

### Statistical methods

A two-tailed Student's t-test was used for between-group comparisons of *in vitro *and flow cytometry data. Tumor growth curves depict median and the error bars interquartile range (25%-75%). Differences were considered significant at the level of *p *< 0.05 (Student's test). The Kaplan-Meier method was used to estimate survival curves from animal studies. Survival curves were compared with the Wilcoxon statistic.

## Results

### ID8 tumor cell killing by topotecan and paclitaxel is phase-dependent

**Figure 1:**
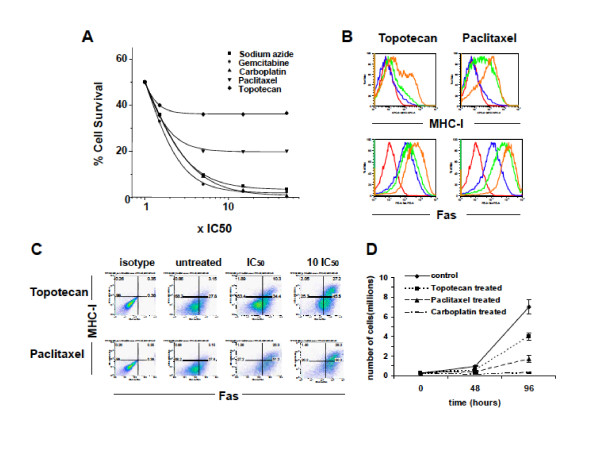
**Topotecan and paclitaxel upregulate MHC-I and Fas on ID8 cells and exert their cytotoxic effects in a phase-specific manner**. **(A) **Cytotoxic effects of paclitaxel, gemcitabine, topotecan, carboplatin or control sodium azide on ID8 tumor cells. ID8 cells were incubated with the chemotherapeutic agents for 6 hours. Survival fraction 42 hours later versus concentration (IC_50 _is used as unit) is shown. Curves are sigmoidal and for the same time of exposure (6 hours) they plateau at a level that depends on the cell cycle specificity. For topotecan and paclitaxel the plateau level is > 30% and > 20% respectively, indicating significant phase specificity. For carboplatin and gemcitabine the sigmoidal curve plateaus at a level < 2.5%. The sigmoidal curves represent the fit of the obtained data to a logistic regression dose response curve. The killing curve of sodium azide (chemical, no interfering with the cell cycle) was used as a negative control. **(B) **Upregulation of MHC-I and Fas on viable ID8 cells treated with topotecan (left) or paclitaxel (right). Cells were exposed to the drugs for 6 hours, washed and incubated in drug free media for 42 hours before harvesting and staining with MHC-I and Fas antibodies. Isotype control (Red); untreated (Blue); Drug concentration inducing approximately 50% killing (IC_50_; Green); Drug concentration corresponding to the plateau of the dose response curve (10 fold IC_50_; Brown). All the histograms depict Annexin-V negative (non apoptotic) cells. **(C) **Dot plot diagrams depicting the upregulation of MHCI and Fas in non-apoptotic tumor cells exposed to Topotecan (upper) and Paclitaxel (lower) at IC_50 _or 10 fold IC_50 _for 6 hours 2 days before. **(D) **Growth curve of sorted MHC-I positive ID8cells following treatment with topotecan, paclitaxel and carboplatin, as indicated. Error bars represent interquartile range (25-75%).

Chemotherapeutic drugs commonly employed against solid tumors such as ovarian cancer, include carboplatin, paclitaxel, topotecan and gemcitabine. Among these, topotecan and paclitaxel are known to be cycle phase-specific and thus their effect is time- rather than dose-dependent. The sensitivity of ID8 mouse ovarian cancer cells to the above drugs was tested *in vitro*. To mimic exposure *in vivo*, where the half life of chemotherapy drugs is short [33,34], cells were exposed to drug for 6 hours and then were followed for an additional 42 hours. Cell count data at 48 hours were fitted to a logistic regression dose-response curve (Figure 1A). The IC_50 _- as calculated from these dose response curves - was used to compare killing curves of the different cytotoxic agents. ID8 cells were killed by all drugs. A characteristic of phase-specific drugs is that their cytotoxicity depends mainly on time of exposure rather than on drug concentration [8,10]. The survival curves of topotecan and paclitaxel reached a plateau at approximately 5-fold the IC_50_, with a substantial fraction of cells surviving in spite of further dose increase; > 35% of the cells survived from topotecan and > 20% of cells survived from paclitaxel. By contrast, gemcitabine and carboplatin exhibited dose-dependent, phase-nonspecific killing *in vitro*.

### Chemotherapeutic drugs induce MHC and Fas expression in tumor cells

It has been suggested that cell damage induced by chemotherapy upregulates the expression of MHC-I or costimulatory molecules (e.g., NKG2D ligands) in tumor cells, sensitizes them to Fas or TRAIL mediated apoptosis [35-42], and renders them susceptible to immune effector cells [3-6]. However, upregulation of immune molecules in tumor cells that have sustained lethal damage from chemotherapy and are destined to die regardless is unlikely to create a suitable basis for synergy between chemotherapy and immunotherapy. Rather, to generate positive interactions, immune effector mechanisms should be able to target tumor cells that are able to escape death from chemotherapy.

We examined whether cells that escape death from time-dependent drugs such as topotecan or paclitaxel upregulate immune molecules implicated in tumor immune attack. We measured the expression of MHC-I and death molecules Fas, TNF-related apoptosis-inducing ligand (TRAIL) and death receptor 6 (DR6) on ID8 cells after short exposure to topotecan or paclitaxel. Dead cells were identified by propidium iodide and apoptotic cells by annexin-V binding. Flow cytometry analysis 42 hrs following exposure to drug showed upregulation of MHC-I and Fas in a subset of viable (propidium iodide-negative and annexin V-negative) ID8 cells (Figure 1B). The upregulation of both MHC-I and Fas in viable cells was dose-dependent and was seen at IC_50 _as well as at concentrations corresponding to the plateau of killing curves (Figures 1B, C). The frequency of non-apoptotic ID8 cells co-expressing MHC-I and Fas after exposure to drugs at 10-fold IC_50 _was seven-fold higher, compared to untreated control cells (27% vs. < 4%, respectively; Figure 1C). Similarly, a fraction of apoptotic (annexin-V positive) cells also upregulated MHC-I and Fas after drug exposure (data not shown). Non-apoptotic ID8 cells did not upregulate expression of the TRAIL receptor DR5, following exposure to paclitaxel or topotecan (not shown). Of note, ID8 cells exposed to carboplatin or gemcitabine also showed dose-dependent upregulation of MHC-I and Fas to a similar degree (not shown). These results show that short exposure to time-dependent (as well as dose-dependent) drugs results in a population of tumor cells that could become potential targets for immune effector cells.

### MHC-I positive tumor cells are capable of re-expanding following exposure to phase-specific drugs

Next, we examined the fate of tumor cells that upregulate MHC-I following short exposure to time-dependent or dose-dependent drugs. We asked whether these cells can undergo proliferation and restore tumor mass. ID8 cells were exposed to topotecan, paclitaxel, carboplatin or gemcitabine at IC_70 _for 6 hours and were then followed in drug-free conditions for 42 hours. Annexin-V negative, MHC-I positive ID8 cells were purified by FACS and were plated in normal media for four additional days. Cells exposed to topotecan proliferated *in vitro *and reached a tumor cell number that was approximately 60% that of a similar starting number of control ID8 cells that were never exposed to cytotoxic agents (Figure 1D). Cells exposed to paclitaxel also proliferated *in vitro *and reached approximately 20% of the number of control untreated ID8 cells (Figure 1D). Thus, following primary exposure to time-dependent drugs, a fraction of annexin-V negative cells that upregulate MHC-I remain viable and maintain their proliferative potential. Similar results were seen with the annexin-V negative, Fas-positive cells following exposure to topotecan or paclitaxel (data not shown). In contrast, annexin-V negative, MHC-I positive ID8 cells treated with carboplatin (Figure 1D) or gemcitabine at IC_70 _(not shown) exhibited no growth *in vitro*. Similarly, annexin-V negative, Fas-positive ID8 cells treated with carboplatin or gemcitabine at IC_70 _exhibited no growth *in vitro *(not shown). Collectively, these results indicate that, although all tested drugs induce a population of tumor cells that expresses MHC-I and Fas and may be targeted by immune effector cells, this population remains viable and is able to restore tumor and thus biologically relevant, only in the case of phase-specific, time-dependent drugs. We thus hypothesized that combination with therapy that boosts immune effector cells benefits selectively time-dependent drugs but not dose-dependent drugs.

### Tumor cells surviving topotecan or paclitaxel are sensitized to effector T cells

**Figure 2:**
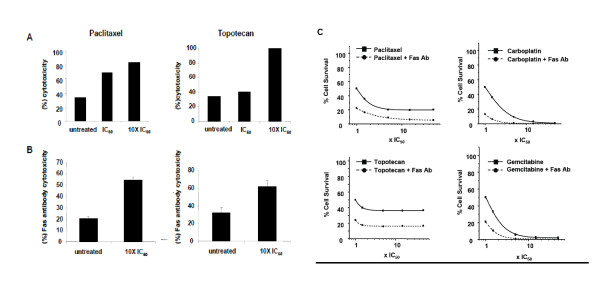
**Tumor cells surviving topotecan or paclitaxel are sensitized to effector T cells**. **(A) **In vitro exposure to paclitaxel or topotecan enhances the sensitivity of ID8-E6E7 cells to activated E7-specific T cells. Bars show cytotoxicity after exposure to 2 different doses of paclitaxel and topotecan (IC_50 _and 10 fold IC_50_) for the same E:T ratio (20:1). Experiments were performed twice with similar results. **(B) **Treatment of ID8 cells with topotecan or paclitaxel sensitizes them to Fas agonistic antibody. ID8 cells (untreated or treated with topotecan or paclitaxel at IC_50 _as described) were incubated with the Fas agonistic antibody and recombinant protein G or with isotype matched antibody and recombinant protein G for 24 hours, harvested, stained with trypan blue and the viable cells were counted (hemocytometer). The bars show the mean level of cytotoxicity and standard errors of three independent experiments. **(C) **The addition of Fas agonistic antibody to paclitaxel (upper left), carboplatin (upper right), topotecan (lower left) or gemcitabine (lower right) *in vitro *targets the resistant ID8 tumor cells and shifts the plateau phase of the dose-response curve for paclitaxel and topotecan downwards. The sigmoid curves represent the fit of the obtained data to a logistic regression dose response curve.

To test the above hypothesis, we used as targets ID8-E6E7 cells, a clonal population of ID8 cells retrovirally transfected to express HPV 16 E6 and E7 oncogenes [24]. ID8-E6E7 cells were treated with topotecan or paclitaxel for 6 hours at IC_60 _or a dose 10 fold the IC_60 _and were harvested 42 hrs later to assess their susceptibility to immune-mediated killing. Effector cells reactive to HPV E7 were expanded from splenocytes of mice previously vaccinated against HPV E7 using E7 synthetic peptide. Prior exposure to paclitaxel or topotecan significantly increased killing of viable ID8-E6E7 cells by splenocytes relative to untreated cells (Figure 2A). Splenocytes from E7-vaccinated mice did not kill control ID8 cells that do not express E7, either at baseline or following exposure to topotecan or paclitaxel (not shown).

To assess whether chemotherapy-treated, viable (annexin-V negative) MHC-I positive and Fas-positive cells are also susceptible to Fas-mediated killing, treated cells were incubated with Fas agonistic antibody. Again, cells treated with time-dependent drugs paclitaxel or topotecan showed increased sensitivity to Fas-induced death (Figure 2B). We tested whether addition to Fas agonistic antibody to chemotherapy increased overall killing of tumor cells. Treatment of ID8 cells with Fas agonistic antibody in combination with paclitaxel or topotecan reduced the surviving fraction by nearly 50% at all concentrations of drug tested (1 to 50 fold the IC_50_), but did not improve carboplatin or gemcitabine treatment at drug concentrations above 10 fold the IC_50 _(Figure 2C). These results support a significant positive interaction between Fas ligation and phase-specific chemotherapeutic drugs.

### IL-18 improves the antitumor effect of time-dependent drugs

**Figure 3:**
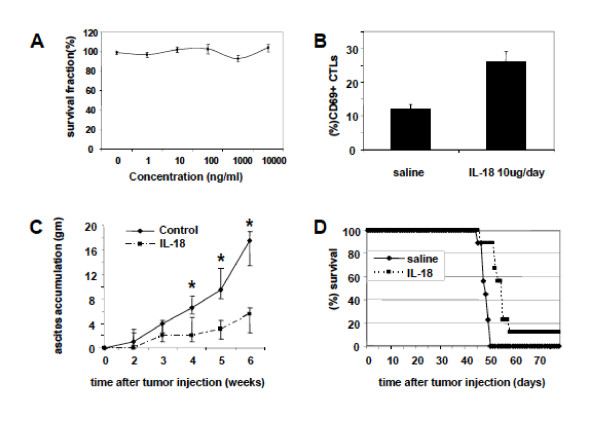
**IL-18 induces activation of T effector cells, restricts ascites accumulation and prolongs survival of ID8-Vegf tumor bearing mice**. **(A) **IL-18 does not have a direct cytotoxic effect on ID8 tumor cells. ID8 tumor cells (5 × 10^5^) were cultured for 48 hours in media containing IL-18 in a wide range of concentrations (0-1000 ng/ml), harvested, and viable cells counted after staining with Trypan blue. Results are means ± SEM of 3 experiments. **(B) **IL-18 induces activation of T effector cells. CD69 expression on CD3^+ ^CD8^+ ^cells isolated from the spleen of ID8-Vegf tumor bearing mice treated with saline or IL-18 (10 μg/day s.c for 20 days, starting 10 days after the tumor challenge). The bars are mean ± SEM of the CD69 expression in CD3^+ ^CD8^+ ^cells from spleens of 5 mice treated with IL-18 as above and 5 mice treated with saline. **(C) **Antitumor properties of IL-18 in C57BL/6 mice. IL-18 significantly restricts ascites accumulation as depicted by increase in the animal weight. The asterisks show data points were the difference between the groups is significant (Student's test, P < 0.05). **(D) **IL-18 significantly prolongs the survival of ID8-Vegf tumor bearing mice (P < 0.05). Control n = 9, IL-18 treated n = 9.

Given the above results *in vitro*, we hypothesized that the addition of IL-18 could selectively increase the efficacy of time-dependent chemotherapeutic drugs such as topotecan or paclitaxel. First, we tested whether IL-18 exerts a direct cytotoxic effect on ID8 or ID8-Vegf tumor cells. No direct cytotoxic effect on ID8 or ID8-Vegf tumor cells was seen *in vitro *(Figure 3A and not shown).

Next, we assessed whether IL-18 therapy induces activation of effector T cells and restricts tumor growth in mice bearing orthotopic i.p. ID8-Vegf tumors. CD3^+ ^CD8^+ ^splenocytes were isolated from mice bearing i.p. ID8-Vegf tumors and treated with 10 μg of IL-18 daily for 20 days, starting 10 days following tumor inoculation. A > 2 fold increase in the frequency of CD3^+ ^CD8^+ ^splenocytes expressing CD69 (12 ± 1.4% for saline *vs*. 26 ± 3.2% for IL-18) was seen after 20 days of IL-18 treatment (Figure 3B). Moreover, IL-18 therapy restricted ascites accumulation, a reliable surrogate of i.p. tumor growth (p < 0.05, Figure 3C), and prolonged the survival of ID8-Vegf tumor-bearing mice (p < 0.05; Figure 3D).

**Figure 4:**
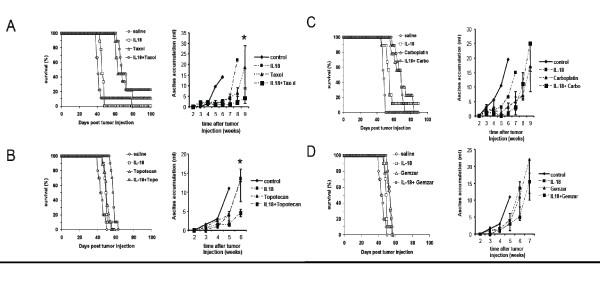
**IL-18 improves only the antitumor effect of time-dependent drugs**. **(A) **Combination therapy with IL-18 improves the survival benefit offered by paclitaxel and topotecan alone and restricts ascites accumulation (left). C57BL/6 mice were injected i.p. with 5 × 10^6 ^ID8-Vegf cells on day 0 and subsequently treated with the indicated types of treatment. Mice treated with the combination of paclitaxel plus IL-18 had a significantly prolonged survival compared to the respective monotherapies (p < 0.01; control n = 23, IL-18 alone n = 22, paclitaxel n = 14, IL-18 plus paclitaxel n = 13). Combination therapy significantly restricted ascites accumulation (right) compared to the respective monotherapies as measured by the increase in the animal weight. The asterisks show data points where the difference in ascites accumulation is significant (Student's test, P < 0.001). **(B) **The combination of topotecan plus IL-18 also significantly prolonged the survival comparing to the respective monotherapies (p < 0.01; control n = 10, IL-18 alone n = 10, topotecan alone n = 9, IL-18 plus paclitaxel n = 10). Combination therapy significantly restricted ascites accumulation (right). The addition of IL-18 to carboplatin **(C) **or gemcitabine **(D) **did not significantly improve survival (left) or restrict ascites accumulation (right). IL-18/carboplatin experiment: Control n = 9, IL-18 n = 9, carboplatin n = 9, IL-18 plus carboplatin n = 9. IL-18/gemcitabine experiment: Control n = 10, IL-18 n = 10, gemcitabine n = 9, IL-18 plus gemcitabine n = 10.

To assess the interactions between IL-18 and time-dependent drugs, we treated animals bearing i.p. ID8-Vegf tumors, with paclitaxel at 15 mg/kg (weekly, four doses), IL-18 (10 μg/day, 40 days), or paclitaxel plus IL-18. Chemotherapy treatment was started 8 days following tumor inoculation and IL-18 treatment was started 2 days later. Control mice were treated with 0.9% saline alone. Paclitaxel alone prolonged median survival by 39% (57 days) while IL-18 alone prolonged median survival by 17% (48 days; Figure 4A). In comparison with paclitaxel monotherapy, the combination of paclitaxel with IL-18 resulted in significant suppression of ascites accumulation (p < 0.001) and better survival (p < 0.01), prolonging median survival by 59% (65 days *vs*. 41days in the control group). Survival at 100 days was 0% in the group treated with paclitaxel; 18% in group treated with IL-18; and 31% in the group treated with the combinatorial therapy; (p < 0.01).

IL-18 administered as above yielded similar results in combination with topotecan at 2.5 mg/kg (every 5 days, 5 doses) (Figure 4B). The combination of IL-18 with topotecan prolonged median survival by 35% (58 day; p < 0.01 relative to topotecan monotherapy), while IL-18 monotherapy prolonged median survival by 14% (49 days) and topotecan alone by 16% (50 days *v*. 43 days in the control group; p < 0.01). The combination of IL-18 with topotecan also reduced ascites accumulation compared to topotecan monotherapy (p < 0.001)

### IL-18 does not improve the antitumor effect of dose-dependent drugs

To reveal the significance of time-dependent drug properties in the interactions between IL-18 and chemotherapy, we tested the combinations of IL-18 with carboplatin or gemcitabine, two chemotherapeutic drugs that kill tumor cells in a dose-dependent manner. Carboplatin was given at 20 mg/kg (weekly, four doses), while gemcitabine was given at 25 mg/kg (every 3 days, 5 doses). IL-18 was given as above. Combination of IL-18 with carboplatin or with gemcitabine did not improve the effect of the chemotherapeutic agent alone either in terms of survival or in terms of ascites accumulation (Figure 4C, D). The median survival in the group treated with carboplatin alone was identical to the group that received the combination therapy. Similarly, the median survival afforded by the combination of IL-18 with gemcitabine was the same as gemcitabine alone. In both cases, IL-18 monotherapy modestly prolonged median survival by 14-15%. There was no significant difference in ascites accumulation between the chemotherapy drugs alone and combination therapy. These results confirm that *in vivo *interactions of IL-18 seen with time-dependent chemotherapy drugs are not seen with dose-dependent drugs.

### Positive interactions between IL-18 and phase-specific chemotherapy drugs are mediated by T cells

**Figure 5:**
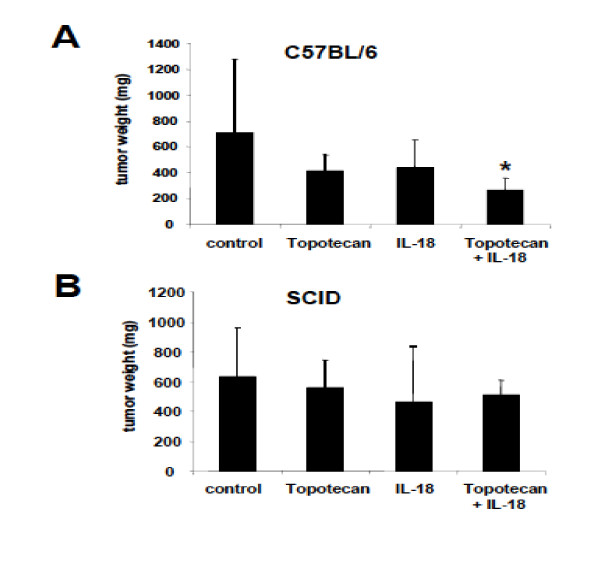
**The antitumor effect of combination IL-18/chemotherapy is T cell dependent**. C57BL/6 **(A) **or C57SCID **(B) **mice were injected s.c. in the flank with ID8-Vegf cells and subsequently treated as described in "Materials and Methods." Mice with no treatment, topotecan alone, IL-18 alone or their combination were sacrificed when control tumors reached a size of approximately 600 to 800 mm^3 ^; all tumors were excised and weighed. Results are medians ± SEM: interquartile range (25%-75%; n = 10). The asterisk indicates the statistically significant difference between experimental and control groups. In the C57BL/6 mice, the topotecan/IL-18 combination treatment significantly restricted the tumor weight relative to the control group (Student's test, p < 0.05), while independent monotherapies did not. In the SCID mice the effect of the combinatorial therapy on the tumor growth was lost with no significant differences between the groups.

Based on the *in vitro *experiments described above, we hypothesized that the observed interactions between IL-18 and time-dependent chemotherapy drugs are mediated by effector T cells which are activated by IL-18 and attack tumor cells surviving the insult of chemotherapy. To test this hypothesis, we evaluated the combination of IL-18 with topotecan in SCID mice lacking T cells. ID8-Vegf cells were injected s.c. in the flank of immunocompetent C57BL/6 or SCID mice. Chemotherapy treatment was started 2 weeks after tumor inoculation, while IL-18 treatment was started 2 days after initiation of chemotherapy. In C57BL/6 mice, combination therapy decreased tumor growth relative to control mice (p < 0.05), while IL-18 or topotecan alone did not have any significant effect (Figure 5A). The median tumor weight in the control group was 714 mg (interquartile range 470-1278); in the topotecan group it was 419 mg (325-540, p = 0.06); in the IL-18 group it was 437.5 mg (240-650, p = 0.066); and in the combination group it was 267 mg (160-360, p = 0.012). In SCID mice, the effect of combination therapy was lost and there were no significant differences between the four experimental arms. The median and interquartile range in the control group were 631 mg (476-962); in the topotecan group were 560 mg (392-742, p = 0.4); in the IL-18 group were 463 mg (317-840, p = 0.14); and in the combination group were 513 mg (292-610, p = 0.13) (Figure 5B).

## Discussion

Although chemotherapy is immunosuppressive and traditional assumption has been that chemotherapy negates the benefits of immunotherapy in cancer, evidence supporting the notion that chemotherapy can be associated with immunotherapy is mounting. For example, chemotherapy can potentially be enhanced by prior vaccine therapy [43]. However, it has been unclear to date whether any chemotherapy drugs are more suitable than others for such chemo-immunotherapy combinations. Whether chemo-immunotherapy combinations can significantly improve the survival benefit obtained with the conventional treatments is under continuing investigation, albeit primarily in empirically designed studies using dose-dependent agents [2,43-48]. Here we characterized the interactions of chemotherapy drugs used commonly against solid tumors with IL-18, a pleiotropic immunostimulatory cytokine. We did not provide direct evidence of phase-selective killing of ID8 cells by the specific chemotherapeutic drugs in this study, because the mechanism of killing of these drugs has been widely documented in the literature so far, including with ovarian cancer cells. For example, in vivo and in vitro studies with topotecan, an S-phase specific drug, demonstrated that a high dose given by bolus injection was less effective than a lower dose given by continuous exposure [49,50]. Our findings indicate for the first time to our knowledge that chemotherapy drugs differ in their ability to interact with immunotherapy, depending on their mechanism of action. Time-dependent (phase-specific) agents, such as topotecan or paclitaxel exhibited positive interactions with immunotherapy *in vivo *while dose-dependent (phase-nonspecific) drugs did not.

Time-dependent drugs target tumor cells in vulnerable phases of the cell cycle, and the fraction of tumor cells killed depends mainly on time of exposure rather than on drug dose [8,10]. Given that the time of *in vivo *exposure to chemotherapy drugs administered in conventional formulations is limited by their short half-life [35,36], a large fraction of tumor cells takes up drug but survives the insult. Our in vitro studies indicate that although these cells remain viable and can reconstitute the tumor, chemotherapy exposes a novel "Achilles' hill" on them. The fraction of viable tumor cells surviving time-dependent chemotherapy exposure showed increased expression of MHC-I and Fas and increased sensitivity to cytotoxic lymphocytes and Fas agonistic antibody. Although MHC-I and Fas were upregulated also in tumor cells treated with phase-nonspecific drugs such as carboplatin and gemcitabine, these cells were all apoptotic and had no tumorigenic potential. The central role of the immune system, particularly effector T cells, in expanding the efficacy of time-dependent chemotherapy was confirmed by the abrogation of the IL-18 and topotecan interaction in tumor-bearing SCID mice lacking T cells. We cannot exclude contribution of other effector cell types, but we expect T cells to be a significant part of the positive interactions between phase-specific chemotherapy drugs and IL-18 in vivo in the mouse and in the human. Thus, an intact immune system is required to observe the therapeutic effects of topotecan and our studies provide novel insights in the complex interactions between chemotherapy and immune therapy. Although the notion that chemotherapy upregulates MHC-I and Fas on tumor cells is not novel, the notion that this becomes biologically and clinically relevant particularly for phase-specific drugs is novel and generates new opportunities for the rational design of chemo-immunotherapy combinations.

In this study we specifically focused on whether, and which, chemotherapy agents sensitize tumor cells to immune effector mechanisms; we thus tried to mainly explore the ability of drugs to render tumor cells more susceptible to immunotherapy with IL-18, and did not investigate each drug's overall effects on the immune system. Additional interactions are possible between chemotherapy and immune therapy. Cytotoxic drugs, when combined with immunotherapy, have the potential to provide a variety of agonistic effects to overcome the multiple barriers to natural antitumor responses. First, chemotherapy can diminish the impact of tumor immunoregulatory factors by reducing tumor burden. For example, during monitoring of T cell responses to antigenic epitopes of cytomegalovirus, EBV, and influenza in advanced ovarian carcinoma patients, it was found that CD8^+ ^T cell responses were significantly lower in patients with high levels of CA125 than in those with low CA125 levels. Furthermore prospective monitoring before, during, and after first-line carboplatin/paclitaxel chemotherapy revealed that CD8^+ ^T cell responses were restored by chemotherapy but only in patients in remission, while patients with progressive disease did not show improvement of CD8^+ ^T cell responses [51]. In addition, chemotherapy can directly eliminate immunosuppressive cells such as regulatory T cells and/or myeloid suppressor cells; deplete host cells that compete with tumor antigen-specific T cells for homeostatic cytokines required for T cell proliferation and survival; and/or promote robust activation of professional antigen presenting cells [52]. Further, cytotoxic agents can modify the tumor microenvironment to favor anti-tumor responses through the promotion of tumor antigen processing and presentation, increased antigen uptake and improved homing of immune cells to tumor [53]. Specifically for gemcitabine, in addition to its apoptotic effects, it promotes the cell-mediated immune response over the humoral immune response by selectively inhibiting B-cell proliferation [54], decreasing memory T cells, and promoting the activation of naive T cells [55] and function of CD8+ T cells [56]. Immunopotentiation is also achieved in part by the inhibitory effects of gemcitabine on myeloid-derived suppressor cells [57]. These mechanisms were recently reviewed by us [58]. Lastly, chemotherapy can induce anti-angiogenic effects [59-61], which could potentially further enhance T cell homing to tumors.

Chemotherapy in ovarian cancer patients has been shown to temporarily reverse immunosuppression, and decrease the proportion of regulatory T cells. Likewise, it increases the percentage of IFN-γ secreting CD8^+ ^cells 12-14 days after administration, thus offering a "window" period for the use of immunotherapy, maximizing the tumor killing effect of both modalities [62]. On the other hand, simultaneous administration of immunotherapy combined with standard chemotherapy, could also be beneficial to ovarian cancer patients and better than a week-delayed schedule [63]. Thereby, not only the choice of the active drug but also the timing of immunotherapy is crucial for optimal results and this should be elucidated in future studies. The observed lack of interaction of gemcitabine with IL-18 in this study could be attributed, not only to the drug's mechanism of action, but also to the specific pharmacologic properties of IL-18. Our results could be affected by the relative sensitivity of tumor cells to each chemotherapy drug, and tumors with increased sensitivity could show different interactions. However, increased sensitivity to the chemotherapy drug does not necessarily mean that interactions with IL-18 would be more pronounced. Similarly, reduced sensitivity to the drug should not preclude interactions to be seen.

## Conclusions

Our results support the concept that immunotherapy can be used to increase the killing effect of chemotherapy. Tumor immunotherapy with IL-18 can significantly augment the killing fraction of phase-specific chemotherapeutic drugs and provide survival benefit. Given that phase specificity is an inherent characteristic of several chemotherapeutic drugs used in a variety of cancers and a major factor that leads to treatment failures, the potential use of immunotherapy as a means to increase the killing fraction of phase dependent drugs, especially in tumors with low mitotic fraction, needs clinical testing. The safety profile of IL-18 and its positive interactions with select anticancer chemotherapeutic agents strongly supports the clinical investigation of this combinatorial approach. Additionally, in order to improve the positive interaction effect of IL-18 and phase-specific chemotherapeutic drugs, different dosing schedules and ways of drug delivery (e.g. liposomal formulations, protein-bound particles, or nanoparticles) should be used in future studies.

## Competing interests

The authors declare that they have no competing interests.

## Authors' contributions

IA carried out the in vitro and in vivo studies and drafted portions of the manuscript. AF, CC, FB and SA contributed materials and assisted in various in vitro and vivo experiments. MT helped in manuscript revision and discussion. ZJ and CHJ participated in the design of the study and data discussion and interpretation. DJP helped to draft the manuscript and supervised statistical analyses. GC designed the study, supervised all steps in execution and wrote the manuscript. All authors read and approved the final manuscript.

## Acknowledgements and Funding

This study was supported by research funding from GlaxoSmithKline and NCI P01-CA83638 SPORE in Ovarian Cancer grant.

## References

[B1] Correale P Cusi MG Tsang KY Del Vecchio MT Marsili S Placa ML Intrivici C Aquino A Micheli L Nencini C Chemo-immunotherapy of metastatic colorectal carcinoma with gemcitabine plus FOLFOX 4 followed by subcutaneous granulocyte macrophage colony-stimulating factor and interleukin-2 induces strong immunologic and antitumor activity in metastatic colon cancer patients J Clin Oncol 2005 23 35 8950 8 10.1200/JCO.2005.12.147 16061910

[B2] Bajetta E Del Vecchio M Nova P Fusi A Daponte A Sertoli MR Queirolo P Taveggia P Bernengo MG Legha SS Multicenter phase III randomized trial of polychemotherapy (CVD regimen) versus the same chemotherapy (CT) plus subcutaneous interleukin-2 and interferon-alpha2b in metastatic melanoma Ann Oncol 2006 17 4 571 7 10.1093/annonc/mdl007 16469753

[B3] Gasser S Orsulic S Brown EJ Raulet DH The DNA damage pathway regulates innate immune system ligands of the NKG2D receptor Nature 2005 436 7054 1186 90 10.1038/nature03884 15995699 PMC1352168

[B4] Gasser S Raulet DH The DNA damage response arouses the immune system Cancer Res 2006 66 8 3959 62 10.1158/0008-5472.CAN-05-4603 16618710

[B5] Groh V Bahram S Bauer S Herman A Beauchamp M Spies T Cell stress-regulated human major histocompatibility complex class I gene expressed in gastrointestinal epithelium Proc Natl Acad Sci USA 1996 93 22 12445 50 10.1073/pnas.93.22.12445 8901601 PMC38011

[B6] Ohtsukasa S Okabe S Yamashita H Iwai T Sugihara K Increased expression of CEA and MHC class I in colorectal cancer cell lines exposed to chemotherapy drugs J Cancer Res Clin Oncol 2003 129 12 719 26 10.1007/s00432-003-0492-0 14564514 PMC12161929

[B7] Durand RE Vanderbyl SL Tumor resistance to therapy: a genetic or kinetic problem? Cancer Commun 1989 1 5 277 83 2702035 10.3727/095535489820874869

[B8] Gardner SN A mechanistic, predictive model of dose-response curves for cell cycle phase-specific and -nonspecific drugs Cancer Res 2000 60 5 1417 25 10728708

[B9] Gieseler F Rudolph P Kloeppel G Foelsch UR Resistance mechanisms of gastrointestinal cancers: why does conventional chemotherapy fail? Int J Colorectal Dis 2003 18 6 470 80 10.1007/s00384-003-0496-x 12774240

[B10] Levasseur LM Slocum HK Rustum YM Greco WR Modeling of the time-dependency of in vitro drug cytotoxicity and resistance Cancer Res 1998 58 24 5749 61 9865733

[B11] Gracie JA Robertson SE McInnes IB Interleukin-18 J Leukoc Biol 2003 73 2 213 24 10.1189/jlb.0602313 12554798

[B12] Jonak ZL Trulli S Maier C McCabe FL Kirkpatrick R Johanson K Ho YS Elefante L Chen YJ Herzyk D High-dose recombinant interleukin-18 induces an effective Th1 immune response to murine MOPC-315 plasmacytoma J Immunother 2002 25 Suppl 1 S20 7 12048347 10.1097/00002371-200203001-00004

[B13] Herzyk DJ Soos JM Maier CC Gore ER Narayanan PK Nadwodny KL Liu S Jonak ZL Bugelski PJ Immunopharmacology of recombinant human interleukin-18 in non-human primates Cytokine 2002 20 1 38 48 10.1006/cyto.2002.1978 12441145

[B14] Robertson MJ Mier JW Logan T Atkins M Koon H Koch KM Kathman S Pandite LN Oei C Kirby LC Clinical and biological effects of recombinant human interleukin-18 administered by intravenous infusion to patients with advanced cancer Clin Cancer Res 2006 12 14 Pt 1 4265 73 16857801 10.1158/1078-0432.CCR-06-0121

[B15] Robertson MJ Kirkwood JM Logan TF Koch KM Kathman S Kirby LC Bell WN Thurmond LM Weisenbach J Dar MM A dose-escalation study of recombinant human interleukin-18 using two different schedules of administration in patients with cancer Clin Cancer Res 2008 14 11 3462 9 10.1158/1078-0432.CCR-07-4740 18519778 PMC8603216

[B16] Horwitz SB Taxol (paclitaxel): mechanisms of action Ann Oncol 1994 5 Suppl 6 S3 6 7865431

[B17] Lorusso D Pietragalla A Mainenti S Masciullo V Di Vagno G Scambia G Review role of topotecan in gynaecological cancers: current indications and perspectives Crit Rev Oncol Hematol 2010 74 3 163 74 10.1016/j.critrevonc.2009.08.001 19766512

[B18] Duffull SB Robinson BA Clinical Pharmacokinetics and Dose Optimisation of Carboplatin Clin Pharmacokinet 1997 33 3 161 83 10.2165/00003088-199733030-00002 9314610

[B19] Rockwell S Grindey GB Effect of 2',2'-difluorodeoxycytidine on the viability and radiosensitivity of EMT6 cells in vitro Oncol Res 1992 4 4-5 151 5 1504375

[B20] Ruiz van Haperen VW Veerman G Vermorken JB Peters GJ 2',2'-Difluoro-deoxycytidine (gemcitabine) incorporation into RNA and DNA of tumour cell lines Biochem Pharmacol 1993 46 4 762 6 10.1016/0006-2952(93)90566-F 8363650

[B21] Mini E Nobili S Caciagli B Landini I Mazzei T Cellular pharmacology of gemcitabine Ann Oncol 2006 17 Suppl 5 v7 12 16807468 10.1093/annonc/mdj941

[B22] Zhang L Yang N Garcia JR Mohamed A Benencia F Rubin SC Allman D Coukos G Generation of a syngeneic mouse model to study the effects of vascular endothelial growth factor in ovarian carcinoma Am J Pathol 2002 161 6 2295 309 10.1016/S0002-9440(10)64505-1 12466143 PMC1850900

[B23] Roby KF Taylor CC Sweetwood JP Cheng Y Pace JL Tawfik O Persons DL Smith PG Terranova PF Development of a syngeneic mouse model for events related to ovarian cancer Carcinogenesis 2000 21 4 585 91 10.1093/carcin/21.4.585 10753190

[B24] Benencia F Courreges MC Coukos G Whole tumor antigen vaccination using dendritic cells: comparison of RNA electroporation and pulsing with UV-irradiated tumor cells J Transl Med 2008 6 21 10.1186/1479-5876-6-21 18445282 PMC2408561

[B25] Halbert CL Demers GW Galloway DA The E7 gene of human papillomavirus type 16 is sufficient for immortalization of human epithelial cells Journal of virology 1991 65 1 473 8 1845902 10.1128/jvi.65.1.473-478.1991 PMC240541

[B26] Bergman AM Eijk PP Ruiz van Haperen VW Smid K Veerman G Hubeek I van den Ijssel P Ylstra B Peters GJ In vivo induction of resistance to gemcitabine results in increased expression of ribonucleotide reductase subunit M1 as the major determinant Cancer Res 2005 65 20 9510 6 10.1158/0008-5472.CAN-05-0989 16230416

[B27] De Cesare M Zunino F Pace S Pisano C Pratesi G Efficacy and toxicity profile of oral topotecan in a panel of human tumour xenografts Eur J Cancer 2000 36 12 1558 64 10.1016/S0959-8049(00)00141-6 10930804

[B28] Mangold G Dexter D Juniewicz P Rake J Von Hoff D Evaluation of paclitaxel-carboplatin-tirapazamine combinations in MV-522 human lung carcinoma xenograft model ASCO Annual Meeting 1997 Abstract

[B29] Travis EL Bucci L Fang MZ Residual damage in mouse lungs at long intervals after cyclophosphamide treatment Cancer Res 1990 50 7 2139 45 2317805

[B30] Wiedenmann N Valdecanas D Hunter N Hyde S Buchholz TA Milas L Mason KA 130-nm albumin-bound paclitaxel enhances tumor radiocurability and therapeutic gain Clin Cancer Res 2007 13 6 1868 74 10.1158/1078-0432.CCR-06-2534 17363543

[B31] Euhus DM Hudd C LaRegina MC Johnson FE Tumor measurement in the nude mouse J Surg Oncol 1986 31 4 229 34 10.1002/jso.2930310402 3724177

[B32] Peng X Treml J Patterson Y Adjuvant properties of listeriolysin O protein in a DNA vaccination strategy Cancer Immunol Immunother 2007 56 6 797 806 10.1007/s00262-006-0240-9 17102978 PMC4180226

[B33] O'Dwyer PJ LaCreta FP Haas NB Halbherr T Frucht H Goosenberg E Yao KS Clinical, pharmacokinetic and biological studies of topotecan Cancer Chemother Pharmacol 1994 34 Suppl S46 52 8070027 10.1007/BF00684863

[B34] Walle T Walle UK Kumar GN Bhalla KN Taxol metabolism and disposition in cancer patients Drug Metab Dispos 1995 23 4 506 12 7600920

[B35] Ciusani E Croci D Gelati M Calatozzolo C Sciacca F Fumagalli L Balzarotti M Fariselli L Boiardi A Salmaggi A In vitro effects of topotecan and ionizing radiation on TRAIL/Apo2L-mediated apoptosis in malignant glioma J Neurooncol 2005 71 1 19 25 10.1007/s11060-004-9180-4 15719269

[B36] Dejosez M Ramp U Mahotka C Krieg A Walczak H Gabbert HE Gerharz CD Sensitivity to TRAIL/APO-2L-mediated apoptosis in human renal cell carcinomas and its enhancement by topotecan Cell Death Differ 2000 7 11 1127 36 10.1038/sj.cdd.4400746 11139287

[B37] Mattarollo SR Kenna T Nieda M Nicol AJ Chemotherapy pretreatment sensitizes solid tumor-derived cell lines to V alpha 24+ NKT cell-mediated cytotoxicity Int J Cancer 2006 119 7 1630 7 10.1002/ijc.22019 16646079

[B38] Micheau O Solary E Hammann A Martin F Dimanche-Boitrel MT Sensitization of cancer cells treated with cytotoxic drugs to fas-mediated cytotoxicity J Natl Cancer Inst 1997 89 11 783 9 10.1093/jnci/89.11.783 9182976

[B39] Morimoto H Yonehara S Bonavida B Overcoming tumor necrosis factor and drug resistance of human tumor cell lines by combination treatment with anti-Fas antibody and drugs or toxins Cancer Res 1993 53 11 2591 6 7684321

[B40] Straughn JM Jr Oliver PG Zhou T Wang W Alvarez RD Grizzle WE Buchsbaum DJ Anti-tumor activity of TRA-8 anti-death receptor 5 (DR5) monoclonal antibody in combination with chemotherapy and radiation therapy in a cervical cancer model Gynecol Oncol 2006 101 1 46 54 10.1016/j.ygyno.2005.09.053 16271751

[B41] Tomek S Horak P Pribill I Haller G Rössler M Zielinski CC Pils D Krainer M Resistance to TRAIL-induced apoptosis in ovarian cancer cell lines is overcome by co-treatment with cytotoxic drugs Gynecol Oncol 2004 94 1 107 14 10.1016/j.ygyno.2004.04.012 15262127

[B42] Yang S Haluska FG Treatment of melanoma with 5-fluorouracil or dacarbazine in vitro sensitizes cells to antigen-specific CTL lysis through perforin/granzyme- and Fas-mediated pathways J Immunol 2004 172 7 4599 608 15034078 10.4049/jimmunol.172.7.4599

[B43] Antonia SJ Mirza N Fricke I Chiappori A Thompson P Williams N Bepler G Simon G Janssen W Lee JH Combination of p53 cancer vaccine with chemotherapy in patients with extensive stage small cell lung cancer Clin Cancer Res 2006 12 3 Pt 1 878 87 16467102 10.1158/1078-0432.CCR-05-2013

[B44] Atzpodien J Kirchner H Jonas U Bergmann L Schott H Heynemann H Fornara P Loening SA Roigas J Müller SC Interleukin-2- and interferon alfa-2a-based immunochemotherapy in advanced renal cell carcinoma: a Prospectively Randomized Trial of the German Cooperative Renal Carcinoma Chemoimmunotherapy Group (DGCIN) J Clin Oncol 2004 22 7 1188 94 10.1200/JCO.2004.06.155 14981107

[B45] Ishida A Miyazawa T Miyazu Y Iwamoto Y Zaima M Kanoh K Sumiyoshi H Doi M Intrapleural cisplatin and OK432 therapy for malignant pleural effusion caused by non-small cell lung cancer Respirology 2006 11 1 90 7 10.1111/j.1440-1843.2006.00790.x 16423208

[B46] Kasamon YL Flinn IW Grever MR Diehl LF Garrett-Mayer E Goodman SN Lucas MS Byrd JC Phase I study of low-dose interleukin-2, fludarabine, and cyclophosphamide for previously untreated indolent lymphoma and chronic lymphocytic leukemia Clin Cancer Res 2005 11 23 8413 7 10.1158/1078-0432.CCR-05-1612 16322303

[B47] Massacesi C Burattini L Marcucci F Bonsignori M Short communication: the efficacy of fixed dose rate infusion of gemcitabine combined with IFN-alpha2a in patients with advanced refractory renal cell carcinoma J Interferon Cytokine Res 2005 25 3 165 8 10.1089/jir.2005.25.165 15767790

[B48] Parra HS Tixi L Latteri F Bretti S Alloisio M Gravina A Lionetto R Bruzzi P Dani C Rosso R Combined regimen of cisplatin, doxorubicin, and alpha-2b interferon in the treatment of advanced malignant pleural mesothelioma: a Phase II multicenter trial of the Italian Group on Rare Tumors (GITR) and the Italian Lung Cancer Task Force (FONICAP) Cancer 2001 92 3 650 6 10.1002/1097-0142(20010801)92:3<650::AID-CNCR1366>3.0.CO;2-0 11505411

[B49] Hoskins P Eisenhauer E Beare S Roy M Drouin P Stuart G Bryson P Grimshaw R Capstick V Zee B Randomized phase II study of two schedules of topotecan in previously treated patients with ovarian cancer: a National Cancer Institute of Canada Clinical Trials Group study J Clin Oncol 1998 16 6 2233 7 9626225 10.1200/JCO.1998.16.6.2233

[B50] Burris H Kuhn J Johnson R Marshall MH Kuhn JG Hilsenbeck SG Von Hoff DD Activity of topotecan, a new topoisomerase I inhibitor, against human tumor colony-forming units in vitro J Natl Cancer Inst 1992 84 23 1816 20 10.1093/jnci/84.23.1816 1331485

[B51] Coleman S Clayton A Mason MD Jasani B Adams M Tabi Z Recovery of CD8+ T-cell function during systemic chemotherapy in advanced ovarian cancer Cancer Res 2005 65 15 7000 6 10.1158/0008-5472.CAN-04-3792 16061686

[B52] Muranski P Boni A Wrzesinski C Citrin DE Rosenberg SA Childs R Restifo NP Increased intensity lymphodepletion and adoptive immunotherapy--how far can we go? Nat Clin Pract Oncol 2006 3 12 668 81 17139318 10.1038/ncponc0666 PMC1773008

[B53] Zitvogel L Apetoh L Ghiringhelli F Kroemer G Immunological aspects of cancer chemotherapy Nat Rev Immunol 2008 8 1 59 73 10.1038/nri2216 18097448

[B54] Nowak AK Robinson BW Lake RA Gemcitabine exerts a selective effect on the humoral immune response: implications for combination chemo-immunotherapy Cancer Res 2002 62 8 2353 8 11956096

[B55] Plate JM Plate AE Shott S Bograd S Harris JE Effect of gemcitabine on immune cells in subjects with adenocarcinoma of the pancreas Cancer Immunol Immunother 2005 54 9 915 25 10.1007/s00262-004-0638-1 15782312 PMC11034286

[B56] Suzuki E Kapoor V Jassar AS Kaiser LR Albelda SM Gemcitabine selectively eliminates splenic Gr-1+/CD11b+ myeloid suppressor cells in tumor-bearing animals and enhances antitumor immune activity Clin Cancer Res 2005 11 18 6713 21 10.1158/1078-0432.CCR-05-0883 16166452

[B57] Bronte V Apolloni E Cabrelle A Ronca R Serafini P Zamboni P Restifo NP Zanovello P Identification of a CD11b(+)/Gr-1(+)/CD31(+) myeloid progenitor capable of activating or suppressing CD8(+) T cells Blood 2000 96 12 3838 46 11090068 PMC2734459

[B58] Kandalaft LE Singh N Liao JB Facciabene A Berek JS Powell DJ Jr Coukos G The emergence of immunomodulation: combinatorial immunochemotherapy opportunities for the next decade Gynecol Oncol 2010 116 2 222 33 10.1016/j.ygyno.2009.11.001 19959212 PMC3119495

[B59] Browder T Butterfield CE Kraling BM Shi B Marshall B O'Reilly MS Folkman J Antiangiogenic scheduling of chemotherapy improves efficacy against experimental drug-resistant cancer Cancer research 2000 60 7 1878 86 10766175

[B60] Bocci G Nicolaou KC Kerbel RS Protracted low-dose effects on human endothelial cell proliferation and survival in vitro reveal a selective antiangiogenic window for various chemotherapeutic drugs Cancer research 2002 62 23 6938 43 12460910

[B61] Gasparini G Metronomic scheduling: the future of chemotherapy? The lancet oncology 2001 2 12 733 40 10.1016/S1470-2045(01)00587-3 11902515

[B62] Wu X Feng QM Wang Y Shi J Ge HL Di W The immunologic aspects in advanced ovarian cancer patients treated with paclitaxel and carboplatin chemotherapy Cancer Immunol Immunother 2010 59 2 279 91 10.1007/s00262-009-0749-9 19727719 PMC11030086

[B63] Braly P Nicodemus CF Chu C Collins Y Edwards R Gordon A McGuire W Schoonmaker C Whiteside T Smith LM The immune adjuvant properties of front-line carboplatin-paclitaxel: a randomized phase 2 study of alternative schedules of intravenous oregovomab chemoimmunotherapy in advanced ovarian cance J Immunother 2009 32 1 54 65 10.1097/CJI.0b013e31818b3dad 19307994

